# Changes in the Expression of Ras-family Genes in Rats Exposed to Formaldehyde by Inhalation

**DOI:** 10.5487/TR.2008.24.3.201

**Published:** 2008-09-01

**Authors:** Guang-Yong Li, Hye-Young Lee, You-Jin Choi, Mi-Ock Lee, Ho-Sang Shin, Hyeon-Young Kim, Sung-Bae Lee, Byung-Hoon Lee

**Affiliations:** 17 College of Pharmacy and Research Institute of Pharmaceutical Sciences, Seoul National University, San 56-1, Sillim-dong, Gwanak-gu, Seoul, 151-742 Korea; 27 Department of Environmental Education and Abuse Drug Research Center, Kongju National University, Kongju, 314-701 Korea; 37 Chemical Safety and Health Research Center, Occupational Safety & Health Research Institute, Daejeon, 305-380 Korea

**Keywords:** Formaldehyde, Inhalation, Microarray, Biomarker, Ras

## Abstract

Exposure to formaldehyde (FA) is closely associated with adverse health effects such as irritation, inflammation, and squamous cell carcinomas of the nasal cavities. Owing to its rapid metabolism and elimination, exposure to FA does not always result in an increased concentration in blood or urine of animals and humans. Therefore, the development of biomarkers for FA exposure is necessary for risk assessment. In the present study, the effects of FA were investigated on the expression of genes involved in the MAPK pathway in vitro and results confirmed in rats exposed to FA by inhalation. Treatment of Hs 680.Tr human tracheal epithelial cells with FA induced gene expression for PDGFA, TNFSF11, SHC1, and HRAS. HRAS expression was also increased in tracheas of rats exposed to FA In addition, FA exposure induced the expression of RASSF4, a member of the Rasassociation domain family of Ras effectors, in rat tracheas. In conclusion, data showed FA-inducible expression of genes involved in the MAPK pathway occurred and increased expression of HRAS and RASSF4 was noted in rat tracheas subchronically exposed to FA by inhalation. These genes may serve as molecular targets of FA toxicity facilitating the understanding of the toxic mechanism.

## INTRODUCTION

Formaldehyde (FA) is one of the volatile organic chemicals present in indoor air, originating from a variety of sources including building materials, cleaning agents, and household products. The colorless, pungent, and chemically reactive gas induces sensory irritation at relatively low concentration in experimental animals and humans (Kane and Alarie, 1977; Alexandersson *et al.*, 1988). FA has been established as one of the main contributors to sick building syndrome (SBS); the adverse health effects produced by serious indoor air quality problems are numerous but most often include mucosal irritation and nonspecific hypersensitivity (Chang *et al.*, 1993). In vitro studies suggested that FA may induce irritation by increasing the expression of adhesion molecules on human mucosal microvascular endothelial cells, thereby enhancing the adhesiveness of the cells to eosinophils (Kim *et al.*, 2002).

Inhaled FA at greater than 6 ppm induces epithelial cell proliferation, cytogenetic abnormalities, and carcinogenic effects at sites of contact (Naya and Nakanishi, 2005). Non-neoplasmic effect by FA includes squamous metaplasia and hyperplasia in the nasal cavity and trachea (Kamata *et al.*, 1997; Shiba *et al.*, 1984; Maronpot *et al.*, 1986). The nasal cavity is the primary site of carcinogenesis in animals exposed to FA, though the trachea is also susceptible (Kerns *et al.*, 1983). Based on evidence in humans and experimental animals, the 2004 International Agency for Research on Cancer (IARC) working group declared FA to be a human carcinogen (IARC, 2004). The formation of protein and DNA adducts (Heck *et al.*, 1990), the enhancement of DNA-protein cross-links, and mutation of p53 (Shaham *et al.*, 2003) are possible molecular mechanisms underlying FA-induced carcinogenesis, although the details have not been elucidated. FA is metabolized to formic acid by aldehyde dehydrogenase, and formic acid is further oxidized to carbon dioxide. Owing to its rapid metabolism and exhalation, FA does not always accumulate at an increased concentration in blood or urine of animals and humans (Heck *et al*., 1985; Gottschling *et al*., 1984). Therefore, FA crosslinks with proteins and DNA to form serum albumin and DNA adducts have been of special interest because of their potential to predict carcinogenesis (Thrasher *et al*., 1990; Zhong and Que Hee 2004; Shaham *et al*., 2003).

Growth factors and other extracellular mitogenic signals activate ERK-1/2 through the activation of the proto-oncogene Ras, producing diverse biological responses. The possibility that the genes involved in the mitogen-activated protein kinase (MAPK) pathway might be used as biomarkers for FA exposure was recently supported by two independent studies. One study (Feick *et al*., 2006) demonstrated that low-dose exposure of intestinal epithelial cells to FA resulted in the activation of ERK-1/2 and p38 MAPK. The other study reported that alpha-platelet-derived growth factor receptor (PDGFRA) transcription was significantly induced in rats exposed to FA by inhalation (Lee *et al*., 2008). The goals of the present study were to identify molecular targets of FA toxicity for the understanding of the toxic mechanism. To this end, the effects of FA on the expression of genes involved in the MAPK pathway were investigated in vitro and results confirmed in rats exposed to FA by inhalation.

## MATERIALS AND METHODS

***Cells and chemicals.*** Hs 680.Tr human tracheal epithelial cell line (ATCC CRL-7422) was maintained in RPMI-1640 containing 10% fetal bovine serum (FBS) in a 5% $CO_2$ atmosphere at 37°C. The passage number of the cells was less than 30. FA was purchased from Sigma Chemical Co. (St. Louis, MO).

***Measurement of cell viability.*** Cells were seeded at a density of $1 \times 10^5$/well in 96 well plate and incubated with FA (0, 20, 50, 100, and 200 μM) for 4 and 24 h in triplicate. Aqueous solution of FA (37%) was added directly to the incubation media and cell viability was measured using the 3-(4,5-dimethylthiazol-2-yl)-2,5-diphenyltetrazolium bromide (MTT) cell proliferation assay. The concentration required for 50% inhibition of the growth ($IC_{50}$) was determined using the results obtained from 3 independent experiments by nonlinear regression analysis using the GraphPad PRISM™ statistics software package (Ver.2.0; San Diego, CA).

***Semiquatitative reverse-transcriptase polymerase chain reaction (RT-PCR).*** Total RNA was prepared using the Easy-Blue™ Total RNA Extraction Kit (Intron Biotech, Korea) and single-strand cDNA was synthesized from the RNA in a reaction mixture containing random hexamers and Superscript II reverse transcriptase (Invitrogen, Carlsbad, CA). Gene-specific primers designed using Oligo 6.0 software (Molecular Biology Insights, Cascade, CO) were used (Table 1). RT-PCR was performed with gene specific primers using Eppendorf Mastcycler. To ensure that the PCR products are quantitated from the linear portion of the amplification curve using the discovery series Quantity One 1-D Analysis software (Bio-Rad). The amplification condition is presented in Table 1.

***Animals.*** Specific pathogen-free male SD rats (6 weeks old, weighing 180~200 g) were purchased from Jung-Ang Laboratory Animals (Seoul, Korea) and were individually housed in stainless-steel wire cages and acclimated to a 12 hr light/ dark cycle. The rats were fed a standard diet (Laboratory Rodent Diet 5001, PMI) and tap water *ad libitum* and were placed in an exposure chamber (whole body type, 1.3 $m^3$, Sibata SIS-800DG, Japan). The airflow through the chamber provided 11 air changes per hr and the pressure was adjusted to 100 mgHg negative pressure. The tempera-

**Table 1.** Gene-specific primer sequences used in RT-PCR analysis

| Gene | NCBI RefSeq | Forward primer (5'-3') | Reverse primer (5'-3') | TM | Cycle |
|---|---|---|---|---|---|
| PDGFA (rat) | NM_012801.1 | TGTAACACCAGCAGCGTCAA | ACATCCGTCTCCTCC*TCCC* | 54 | 28 |
| (human) | NM_033023.3 | CCCGCAGTCAGATCCACA | GCAGCGTTTCACCTCCAC | 56 | 28 |
| TNFSF11 (rat) | NM_057149.1 | CATCGGGTTCCCATAAAGTC | TGAAGCAAATGTTGGCGTA | 50 | 28 |
| (human) | NM_003701.2 | CGTCGCCCTGTTCTTCTA | GGGATGTCGGTGGCATTA | 54 | 28 |
| SHC1 (rat) | NM_053517.1 | GGGTTGCGTGGAGGTCTTA | CAGTCTGCTGCCATGAGGTT | 56 | 28 |
| (human) | NM_003029.3 | GGAAGAGCCACCTGACC | GGCACGCGAAGAGCATC | 56 | 28 |
| HRAS (rat) | XM_001061671.1 | TGCCATCAACAACACCAAGTC | AGTTTATGCTGCCGAATCTCAC | 54 | 30 |
| (human) | NM_005343.2 | TGCCATCAACAACAACAAGT | TCTCATCAGGAGGGTTCAGC | 52 | 30 |
| RASSF4 (rat) | NM_001024275.1 | CTTATGCGGACAAAGAGT | TGGGTGGTCATAGTGCTG | 49 | 28 |

ture and humidity were maintained at 23 ± 3°C and 50~60%, respectively. The experimental protocol was approved by the Committee for the Care and Use of Laboratory Animals at this institute by the Korean Academy for Medical Sciences.

***Exposure to FA.*** The rats were randomly assigned to three groups (vehicle control, low FA, high FA) and exposed to the FA aerosols for 6 hr/day, 5 days/week in the inhalation chamber for two weeks. FA aerosol was generated using a mist generating system (NB-2N & DM-800, Sibata Co., Japan) equipped with a jet spray. The concentration of FA in the inhalation chamber was monitored several times a day throughout the experiment by gas chromatography (Lee *et al*., 2008). The mean concentration of FA was 3.1 ± 0.9 ppm (low FA) and 38.1 ± 7.2 ppm (high FA). Immediately after cessation of the exposure, tracheas were removed and placed in RNAlater (Ambion Inc., Austin TX) for RT-PCR from 5 rats in each group.

***Statistical analysis.*** All data were expressed as mean ± SD. Statistical analysis was performed using Student's *t* test. Differences between groups were considered to be statistically significant at $p < 0.05$.

## RESULTS

***Cytotoxicity of FA.*** To establish the optimal concentration of FA for RT-PCR experiments, Hs 680.Tr cells were incubated with medium containing FA (0, 20, 50, 100,or 200 μM) and then cell viability determined using the MTT colorimetric assay. The concentrations of FA that induced the half-maximal cytotoxic effect ($IC_{50}$) were 99.6 ± 1.1 μM at 4 hr and 69 ± 1.1 μM at 24 hr.

***Expression of genes involved in the MAPK pathway in Hs 680.Tr cells.*** To minimize the fixative effect of FA, which increases with concentration and time, changes in gene expression were analyzed after 4 and 24 hr incubation with either 10 or 100 μM FA. The genes studied were PDGFA, TNFSF11, SHC1, and HRAS, which are regarded as components of the MAPK pathway representing extracellular stimuli, adaptors, and effector proteins. Although the expression pattern differed among the genes, the expression level of all 4 genes was increased in Hs 680.Tr cells following FA treatment. PDGFA and TNFSF11 expression was elevated only at high FA concentration after 4 and 24 hr, respectively. Transcription of SHC1 was induced in nearly every treatment condition, whereas, HRAS transcription was up-regulated at low FA concentrations at both time points (Fig. 1).

***Identification of genes induced by FA exposure in rat trachea.*** Based on the in vitro results, the expression of these genes was examined in tracheas obtained from rats exposed to low (3.1 ± 0.9 ppm) or high concentrations (38.1 ± 7.2 ppm) of FA by inhalation. The expression levels of PDGFA, SHC1, and TNFSF11 were not affected; however, the level of HRAS mRNA was increased in a dose-dependent manner, with a statistically significant change at the high FA concentration compared with control (Fig. 2). To test whether other members of the Ras-association domain family of Ras effectors are also affected by FA exposure, RT-PCR was performed with a gene-specific primer for RASSF4, which synergizes with K-Ras to induce apoptotic cell

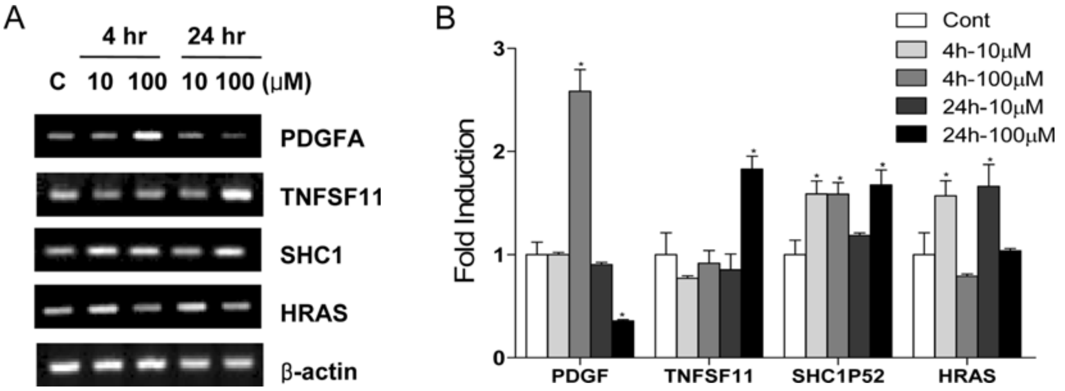

**Fig. 1.** Induction of genes following exposure to FA *in vitro*. Hs 680.Tr cells were incubated with FA (10 and 100 μM) for 4 or 24 hr, and the expression levels of the indicated genes were determined by RT-PCR. Representatives of three independent assays with similar results are shown (A). The densities of the PCR product bands on a gel were determined using an image analysis system. The values were normalized to β-actin and expressed as fold induction relative to control. Each bar represents mean ± SD of three independent experiments (B).

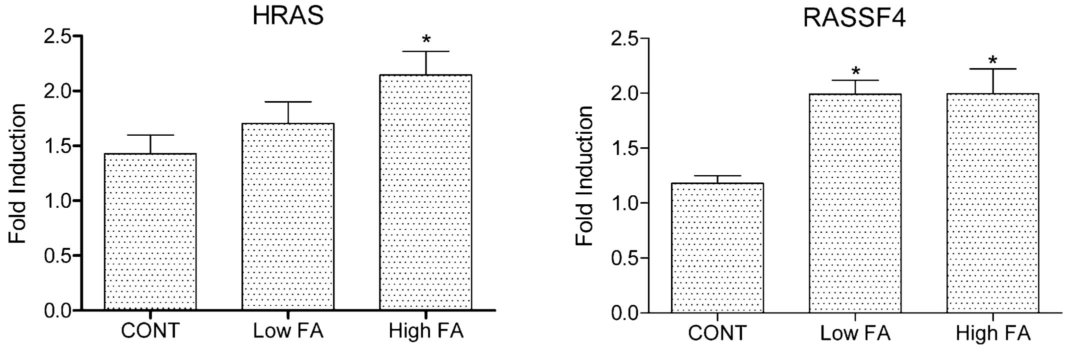

**Fig. 2.** Induction of genes following exposure to FA *in vivo*. Fifteen rats were divided into three groups (control, low FA, high FA) and exposed to FA aerosols for 6 hr/day, 5 days/week for 2 weeks, in the inhalation chamber. The expression levels of the indicated genes were determined by RT-PCR, and the densities of PCR product bands on gels were determined using an image analysis system. The values were normalized to β-actin and expressed as fold induction relative to control. Data shown are the mean ± SD (n = 5). $^*p < 0.05$ compared to control.

death. As shown in Fig. 2, the expression of RASSF4 was significantly up-regulated in FA-treated rats. A meaningful pattern of change between the two genes was noted.

## DISCUSSION

Environmental and industrial health experts have focused more attention on FA, primarily owing to its close association with adverse health effects ranging from irritation and inflammation (Kane and Alarie, 1977; Alexandersson *et al.*, 1988) to squamous cell carcinomas of the nasal cavities (Kerns *et al.*, 1983), depending on the concentration and duration of exposure. There is increasing evidence supporting a correlation between exposure to FA and development of SBS, which is generally characterized by mucosal irritation and nonspecific hypersensitivity (Chang *et al.*, 1993). Although the specific cause of SBS has not yet been identified, the accumulation of chemical and biological contaminants from indoor sources due to inadequate ventilation is regarded as one of the main factors responsible for SBS. Among the many chemicals present in indoor air, volatile organic compounds such as FA are well known to produce SBS (Caldas *et al.*, 1994; Harada *et al.*, 2007).

Despite widespread FA contamination of indoor air and serious adverse health effects associated with FA, there are few reports on biomarkers for FA exposure. The measurement of FA or its metabolites in body fluids does not accurately reflect the extent of FA exposure owing to the highly reactive nature of FA; this is especially true at low exposure levels (Gottschling *et al.*, 1984). For this reason, the reaction of FA with macromolecules and the formation of DNA-protein cross-links in peripheral blood lymphocytes have been used as surrogate measures for FA exposure (Shaham *et al.*, 2003). However, DNA-protein cross-links are not specific for FA; they also form following exposure to many other environmental chemicals or ionizing radiation (Al-Nabulsi and Wheeler, 1999; Chakrabarti *et al.*, 2001). Several scientists used genomics and proteomics to identify potential biomarkers for FA exposure. Genes and proteins make reliable biomarkers because they are sensitive enough to detect exposure to low levels of FA and provide insight as to the mechanism of FA-induced toxicity. The expression patterns of some genes was shown to be correlated with exposure to FA, and this was further demonstrated in a study of residents in a new building who were thought to have been exposed to FA (Im *et al.*, 2006; Li *et al.*, 2007; Lee *et al.*, 2008a, b).

A mechanism-based biomarker for chemical exposure can be defined as a measure that characterizes a causal process along the continuum from chemical exposure to adverse event (Danhoff *et al.*, 2005). Research on the mechanism of a chemical of interest might reveal such a biomarker. The mechanisms of FA-induced SBS and cancer have been partly described. Increased numbers of CD26- and IL-2-positive cells, B cells, and autoantibodies (Thrasher *et al.*, 1990) as well as the induction of adhesion molecules (Kim *et al.*, 2002) have been reported in SBS. A possible mode of action in nasopharyngeal cancer is thought to involve DNA adducts and DNA-protein cross-links that have been observed at the site of first contact with FA (Casanova *et al.*, 1989; Monticello *et al.*, 1996). The activation of MAPK in epithelial cells is also thought to play a role in FA-induced carcinogenesis (Feick *et al.*, 2006). Any of these processes may provide candidates for a mechanism-based biomarker for FA exposure. In the present study, we hypothesized that stimuli, adaptor, or effector molecules belonging to the MAPK pathway could be efficient biomarkers for FA exposure. To verify the hypothesis, the expression of PDGFA, TNFSF11, SHC1, and HRAS mRNA was measured in a human tracheal cell line and in tracheal tissue from rats, after exposure to FA. All four genes were induced *in vitro*, but only HRAS expression was altered *in vivo*. The expression of RASSF4, a Ras effector protein, was also increased by FA exposure. The comparison between the results from cultured cells and rats provides some interesting differences which might be ascribed to many factors including the mode of application, the dose and duration of exposure, the species difference between human cells and rats, and, above all, the intrinsic differences between *in vitro* and *in vivo* experiment.

H-Ras is a small G protein that acts as a molecular switch to control signaling pathways that regulate normal cell growth and malignant transformation (Takai *et al.*, 2001). Ras exerts its biological effects by interacting with multiple effectors to activate diverse signaling pathways. The serine/threonine kinase Raf is one of the downstream effectors activated by Ras; which in turn activates a kinase cascade such as MAPK (Leevers *et al.*, 1994). Mutations in Ras, the over-expression of pathway receptors, and the over-expression of Ras genes result in persistent activation of the Ras-MAPK pathway (Calipel *et al.*, 2003). Evidence suggests that the growth-promoting activity of Ras is mediated by the Raf/MAPK pathway. Mansour *et al.* (1994) demonstrated that cells with constitutively active MAPK kinase formed transformed foci and were highly tumorigenic in nude mice. Conversely, the inhibition of MAP kinases by MAP kinase phosphatase was shown to block the mitogenic and oncogenic effects of Ras (Sun *et al.*, 1994).

In the present study, FA-inducible genes involved in the MAPK pathway were identified and induction of HRAS and RASSF4 demonstrated in tracheas from rats subchronically exposed to FA by inhalation. These genes may serve as molecular targets of FA toxicity facilitating the understanding of the toxic mechanism.

## ACKNOWLEDGEMENTS

This work was supported by the Ministry of Environment as “The Eco-Technopia 21 Project” and by the SRC/ERC program of MOST/KOSEF (R11-2007-107-01001-0).
